# The Chronic Effects of Low- and High-Intensity Resistance Training on Muscular Fitness in Adolescents

**DOI:** 10.1371/journal.pone.0160650

**Published:** 2016-08-10

**Authors:** Ari R. Assunção, Martim Bottaro, João B. Ferreira-Junior, Mikel Izquierdo, Eduardo L. Cadore, Paulo Gentil

**Affiliations:** 1 College of Physical Education, University of Brasília, Brasília, DF, Brazil; 2 Federal Institute of Sudeste of Minas Gerais, Campus Rio Pomba, Rio Pomba, MG, Brazil; 3 Department of Health Sciences, Public University of Navarre, Campus de Tudela, Tudela, Navarre, Spain; 4 School of Physical Education, Federal University of Rio Grande do Sul, PA, RS, Brazil; 5 College of Physical Education, Federal University of Goias, Gôiania, GO, Brazil; Pondicherry Institute of Medical Sciences, INDIA

## Abstract

To compare the effects of high-load, low-repetition maximum (LRM) and low-load, high-repetition maximum (HRM) resistance training regimens on muscular fitness in untrained adolescents. Forty-five untrained adolescents of both sexes (13.7±0.8 years; 161.3±7.5 cm, 56.8±13.4 kg) were randomly assigned into one of three groups: 1) LRM (n = 17): volunteers performed three sets of 4-6-repetition maximum (RM); 2) HRM (n = 16): volunteers performed three sets of 12–15 RM; and 3) control (CON, n = 12). Training was performed two times a week for 9 weeks. After training, there were significant increases in 1 RM chest press (LRM = 14.8% and HRM = 14.2%, p<0.05) and squat (LRM = 26.4% and HRM = 25.7%, p<0.05), with no differences between the LRM and HRM groups (p>0.05). Additionally, muscular endurance increased significantly for the chest press (LRM = 14.5% and HRM = 21.8%, p<0.05) and squat test (LRM = 31.4% and HRM = 32.4%, p<0.05) following resistance training, with no difference between the LRM and HRM groups (p>0.05). These results suggest that both high-load, low-repetition and moderate-load, high-repetition resistance training can be prescribed to improve muscular fitness in untrained adolescents.

## Introduction

Resistance training has been recommended as an important component of training programs, mainly due to its capacity to increase muscular strength and size. Children and adolescents also benefit from resistance training [1, 2], with muscular strength gain increasing with age and maturational status [3]. In addition to hypertrophy and muscle force gains, it has been reported that resistance training can improve important health parameters (i.e., bone mineral density, body composition, and physical fitness capacities) in youths [1, 2, 4–7]. A previous study reported that high muscular strength in adolescence was associated with a 20–35% lower risk of premature mortality due to any cause or cardiovascular disease, independently of other factor, as body mass index or blood pressure [8]. The study also revealed that stronger adolescents were 15–65% less likely to have psychiatric problems and had a 20–30% lower risk of committing suicide. Additionally, resistance training plays an important role in youth who are overweight and obese, since may improve muscle strength, body composition, motor coordination, and self-confidence in this population [1]. Therefore, increasing muscle strength in adolescence seems to be an important health issue, and studies assessing the efficiency of different resistance training protocols in this population are needed.

To optimize gains in muscle strength and mass, professionals who prescribe resistance training regimens must take into account many variables such as volume, loading, exercise choice and order, rest period, velocity, muscle action, and training frequency [9, 10]. Training load and volume have been considered as one of the main variables for youth resistance training [1]. In adults, resistance training programs characterized by high loads and low repetitions have been reported to induce greater force gains compared to programs utilizing low loads and high repetitions [11–17]. However, other studies did not report differences in strength increase between high-load and moderate-load resistance training [18–21]. Interestingly, children appear to respond differently to resistance training. Previous studies have shown similar gains in muscle strength in children after high-repetition maximum (6–10 RM) training compared to low-repetition maximum (13–20 RM) regimens [22–24]. According to the authors, these differences in children are related to neural influences rather than hypertrophic factors, possibly due to the low level of testosterone in children [24].

Another outcome that can be largely influenced by variable selection is muscle endurance. Greatest increases on muscular endurance were observed after training composed of low loads and high repetitions in adults [25, 26]. In addition, greater increases in muscular endurance were obtained by children who performed 13 to 20 RM in comparison with those who performed 6 to 10 RM programs [22–24]. Considering that previous studies suggest that different protocols result in divergent adaptations in muscle strength and endurance [25, 26], it would be important to evaluate the impact of manipulating training intensity and volume in both muscle strength and endurance gains.

The current stance on youth resistance training recommends low volume (1–2 sets and 1–3 repetitions) and low-moderate training intensity (<60% 1 RM) for youth without resistance training experience and, once basic exercise technique is competent, progress to higher intensity and volume (i.e. 2–4 sets of 6–12 repetitions with ≤80% 1 RM) [1]. A recent meta-analysis on children and adolescents reported a positive dose response relationship for intensity in subjects who performed resistance training regimens [27]. According to this study, the minimal exercise intensity for induce gains in motor performance skills in children and adolescents is 50% [27]. However, these recommendations are based primarily on studies in children. Considering that adolescents differ from both children and adults in hormonal status [28] and in the acute responses to resistance training [28, 29] and that the ability to gain muscular strength seems to increase with age and maturational status [1, 3], recommendations based on children and adults may not be optimal for adolescents.

To the best of our knowledge, no study has investigated the effects of different arrangements of load and volume resistance training on strength and muscular endurance adaptations in adolescents; thus, this topic requires further investigation. In addition, investigating this issue could help strength and conditioning trainers to design better training programs for strength training in adolescents. Therefore, the aim of this study was to compare the effects of high-load, low-repetition maximum (LRM) and moderate-load, high-repetition maximum (HRM) resistance training programs on strength and muscular endurance gains in untrained adolescents during the initial adaptation period. Our hypothesis is that: 1) muscular strength gains will be higher in the high-load, low-repetition maximum group, and 2) the gains in muscular endurance will be the same between both training groups.

## Materials and Methods

### Subjects

The participants were from the same institution, the Integrated Center of Physical Activity, a public institution were children and adolescents are introduced and trained in different sports modalities. They have an option to choose one activity to practice during one semester. Adolescents who opted to perform resistance training were invited to join the study.

Sixty adolescents were initially contacted, but forty-five (19 male and 26 female; 13.7 years ± 0.8 years; 161.3 ± 7.5 cm, 56.8 ± 13.4 kg) finished the study. The participants and their parents provided written consent prior to the study. They were informed about the experimental procedures and the benefits and risks of the study before signing a statement of written informed consent. The present study was performed in accordance with the Code of Ethics of the World Medical Association (Declaration of Helsinki), and the College of Health Sciences Ethics Committee of the University of Brasilia granted approval for the study (CAEE 38384514.6.0000.0030). To be included in the study, potential participants had to be 13–15 years old, had to have never taken part in a resistance training program and be free of health problems. All participants were in the Tanner stages 3 and 4. Participants were excluded if they did not attend at least 80% of the training sessions [30]. The participants were asked to not change their nutritional habits (e.g., becoming a vegetarian, restricting caloric intake, or using nutritional supplements or ergogenic substances). Moreover, all adolescents were involved in moderate physical activity (jogging, agility or endurance) for an average of 3 days a week.

### Experimental design

The first three weeks consisted of anthropometric assessment, familiarization with the procedures of the study, and the testing of and re-testing maximum strength (1 RM) and muscular endurance. During familiarization sessions, participants were instructed in how to correctly perform the exercises, and initial load values were obtained. Thereafter, the subjects were randomly divided into three groups by block randomization [31]: 1) a low-repetition resistance training group (LRM, n = 17), which involved 4 to 6 RM of eight resistance exercises; 2) a high-repetition resistance training group (HRM, n = 16), which involved 12 to 15 RM of eight resistance exercises; and 3) a control non-exercise group (CON, n = 12). Initially, 20 participants were assigned for each group; however, three from HRM, four from LRM and eight from CON did not complete the study due to attending less than 80% of the training session or not performing all tests. Both LRM and HRM groups performed the same resistance exercises twice weekly over the course of 9 weeks. The participants of each group trained together in the same resistance training facility (LRM trained from 2PM to 3PM and HRM trained from 3PM to 4PM) and the control group was informed that they would initiate RT classes after the second final evaluations.

Maximum strength (1 RM) and muscular endurance on a barbell chest press and a Smith machine squat were assessed at baseline and post-training. Differences in maximal strength and muscular endurance gains in response to different resistance training regimens were compared among the LRM, HRM and CON groups. Additionally, subjects were asked to visit the laboratory at the same time of the day to avoid circadian influences. They were also instructed not to take medications or supplements during the study period.

### 1 RM test

Maximal strength was determined by assessing 1 RM for the chest press and squat exercises. The 1 RM test was performed on a barbell bench press and on a Smith machine squat. Weight plates starting at 0.5 kg were used to adjust the load. On the testing day, subjects performed a warm-up consisting of 8 repetitions at 40 to 50% of their estimated 1 RM. After a 60 s rest interval, they performed 6 repetitions at 50 to 60% of their estimated 1 RM. Then, each subject had a maximum of five attempts to achieve his or her 1 RM load. The rest interval between attempts was 5 min. Range of motion was controlled for chest press and squat exercises. For chest press exercise, subjects had to touch their chest at the end of the eccentric phase and return to a position with their elbows fully extended at the end of the concentric phase. In addition, their neck, head, shoulders, and hips were kept in contact with the bench throughout the exercise, with their feet on the floor. For squat exercise, subjects had to flex their knees at 90° (0° full extension) at the end of the eccentric phase and return to a position with their knees fully extended at the end of the concentric phase. Subjects received verbal encouragement throughout the test, and the same investigator performed all testing procedures. Test-retest reliability coefficients (ICC) were 0.97 and 0.96 for the 1 RM chest press and squat exercises, respectively.

### Muscular endurance test

Muscular endurance was determined by assessing the number of repetitions prior to failure for the chest press and squat exercises. Prior to the test, subjects performed a warm-up consisting of 10 repetitions at 50% of their baseline 1 RM. Two minutes later, each subject carried out repetitions until failure at 70% of their baseline 1 RM. Each repetition took 1.5 s for concentric and eccentric actions and was controlled by an electronic metronome. The test was finished when subjects were unable to keep up with the metronome pace. Range of motion was controlled for chest press and squat exercises as described above. The participants received verbal encouragement throughout the test, and the same investigator performed all testing procedures. Test-retest reliability coefficients (ICC) were 0.94 and 0.88 for the chest press and squat endurance test, respectively.

### Resistance training protocols

All volunteers in both the LRM and HRM groups performed the same exercises: leg press, knee extension, barbell chest press, dumbbell fly, lat pulldown, seated row, crunches, and leg raises. The LRM group performed 2 sets at 4 to 6 RM, while the HRM group carried out 2 sets at 12 to 15 RM for each exercise. If necessary, loads were adjusted at each set to maintain the designed number of repetitions of each group. For crunches and leg raises, both the LRM and HRM groups performed 2 sets of 15 repetitions. The rest interval was 60 s between sets and 120 s between exercises. Subjects were instructed to perform 2 s for concentric and eccentric muscle actions. Participants were oriented to perform all sets until concentric failure. If necessary, loads were adjusted at each set to maintain the designated number of repetitions. Training was conducted twice a week, with a minimum of 48 h between sessions. Each participant filled out a training log for each training session, containing the loads used and the number of repetitions performed in each set. All training logs were verified by a supervisor following every exercise session. All training sessions were closely supervised by experienced and certified trainers [32]. Moreover, the adolescents were not allowed to perform any extra resistance exercise.

### Statistical analyses

Data are reported as the mean ± standard deviation. The Shapiro-Wilk test was used to assess the data for normal distribution. The data exhibited a normal distribution, so a 3x2 [groups (CON, LRM and HRM) x time (pre- and post-training)] mixed model ANOVA was used to analyze maximal strength and muscular endurance. In the case of significant differences, Tukey’s post hoc test was used. The intraclass correlation coefficient (ICC) was used to measure intra-rater reliability. The significance level was set at P< 0.05. Physical characteristics, baseline maximal strength, and muscular endurance were evaluated using a one-way ANOVA. In the case of significant differences, a Fisher LSD post hoc test was used. The reliability of all measurements was calculated by intraclass correlation coefficient values (ICC) using single values. The significance level was set at P<0.05. In addition, effect size was calculated according to Beck [33], and *d* values were defined as small, medium, and large based on Cohen’s ranges of 0.2, 0.5, and 0.8, respectively.

## Results

Physical characteristics, baseline maximal strength and muscular endurance did not differ among the groups (p>0.05, Table 1). Mean training volume accumulated (sets by repetition by load) over the training period in the bench press was 10,282 ± 3,076 kg for HRM and 6,268 ± 1,949 kg for the LRM. In the leg press, the values were 27,558 ± 7,264 kg and 14,168 ± 3,529 kg for the HRM and LRM, respectively. Values for HRM were higher than for LRM (p<0.05)

**Table 1:** Participants’ physical characteristics of each experimental Group.

	LRM group (10M/7F)	HRM group (7M/9F)	CON group (2M/10F)	P value
Age (years)	13.8 ± 0.9	13.7 ± 0.7	13.7 ± 0.7	0.87
Height (cm)	161.2 ± 7.3	162.2 ± 5.2	160.6 ± 10.1	0.85
Weight (kg)	54.7 ± 18.8	58.6 ± 10.2	57.1 ± 11.2	0.74
1RM Chest press (kg)	31.4 ± 7.1	30.9 ± 7.1	29.3 ± 5.5	0.69
1RM Squat (kg)	61.2 ± 13.1	60.9 ± 10.6	62.7 ± 10.7	0.91
Endurance Chest press (repetitions)	11.4 ± 4.5	10.9 ± 2.3	10.6 ± 4.5	0.98
Endurance Squat (repetitions)	10.3 ± 4.0	11.0 ± 5.9	8.1 ± 1.2	0.33

LRM group: heavy load–low repetition maximum resistance training group. HRM group: moderate load–high repetition maximum resistance training group. CON group: control group. 1RM Chest: maximal strength in the chest press. 1RM Squat: maximal strength in the squat. Endurance Chest: muscular endurance in the chest press. Endurance Squat: muscular endurance in the squat.

**Fig 1:**
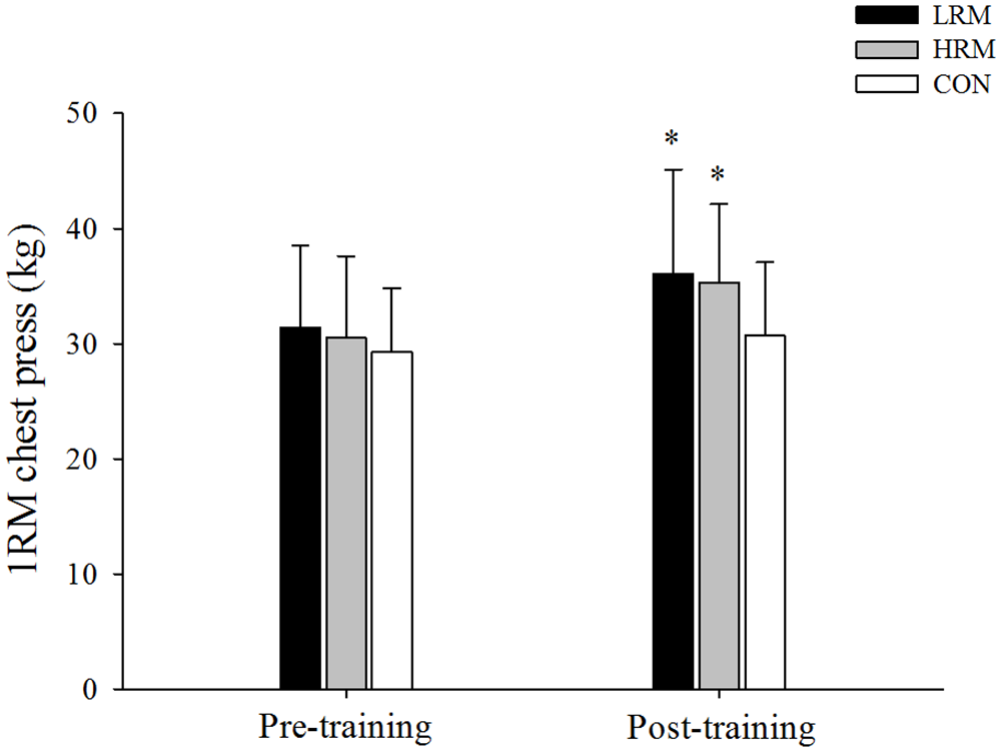
Mean ± SD of chest press maximal strength pre and post 9 week of resistance training. LRM, heavy load–low repetition maximum resistance training group. HRM, moderate load–high repetition maximum resistance training group. CON, control group. (*) p<0.05, higher than pre training.

**Fig 2:**
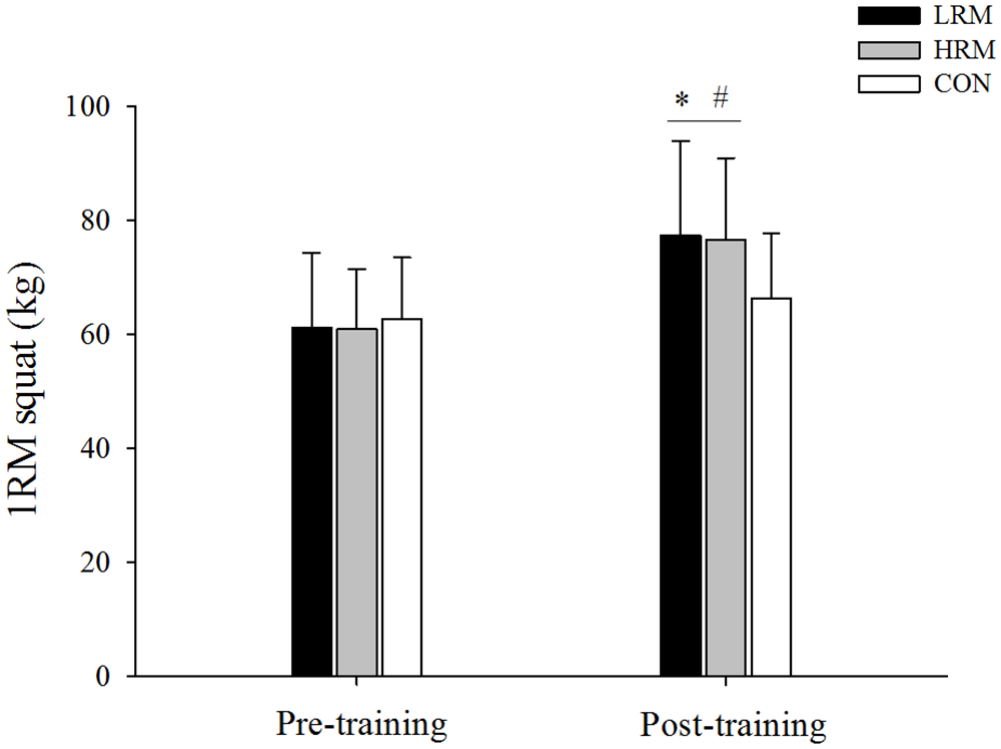
Mean ± SD of squat maximal strength pre and post 9 week of resistance training. LRM, heavy load–low repetition maximum resistance training group. HRM, moderate load–high repetition maximum resistance training group. CON, control group. (*) p<0.05, higher than pre training. (#) p<0.05, higher than CON group.

There was a significant difference in time interaction for chest press (F = 6.4, p = 0.004, 1-β = 0.82, Fig 1) and squat maximal strength (F = 15.2, p<0.001, 1-β = 0.99, Fig 2) by group. After training, there were significant increases in chest press and squat maximal strength for both the LRM (*d* = 0.68 and *d* = 0.77, respectively) and HRM (*d* = 0.69 and *d* = 0.78, respectively) groups (p<0.05), while no change was observed in the CON group (p>0.05). However, there was no difference in chest press and squat maximal strength gains between the LRM and HRM groups (p>0.05). The effect size of LRM and HRM resistance training was moderate for chest press (*f* = 0.68 and *f* = 0.69, respectively) and for squat maximal strength (*d* = 0.77 and *d* = 0.78, respectively). In addition, squat maximal strength post-training was higher in the LRM and HRM groups when compared to the CON group (p<0.05).

**Fig 3:**
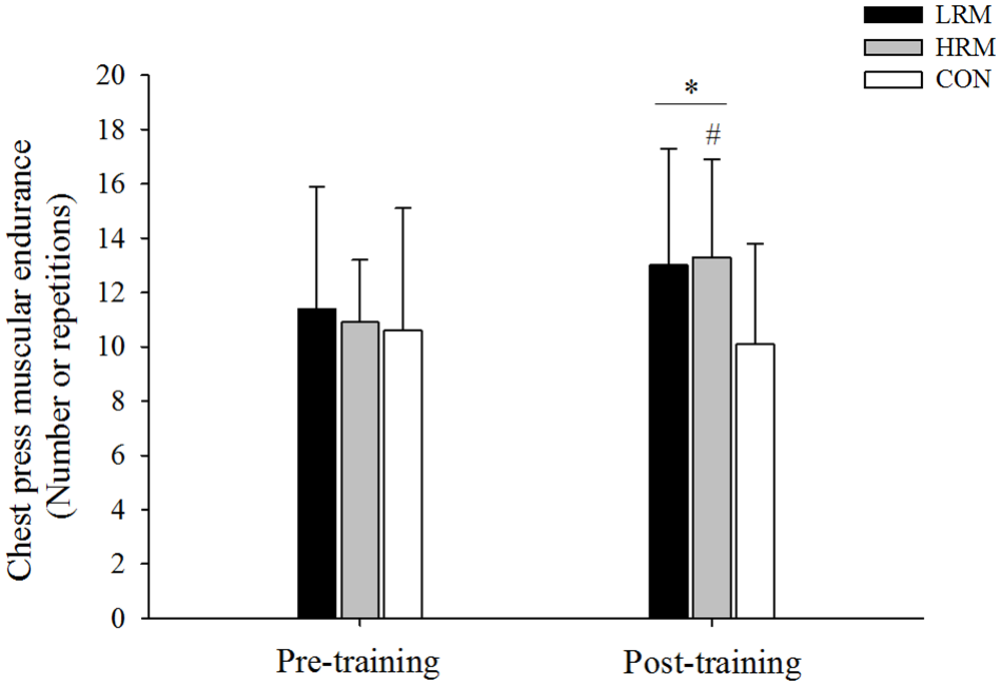
Mean ± SD of chest press muscular endurance pre and post 9 week of resistance training. LRM, heavy load–low repetition maximum resistance training group. HRM, moderate load–high repetition maximum resistance training group. CON, control group. (*) p<0.05, higher than pre training. (#) p<0.05, higher than CON group.

**Fig 4:**
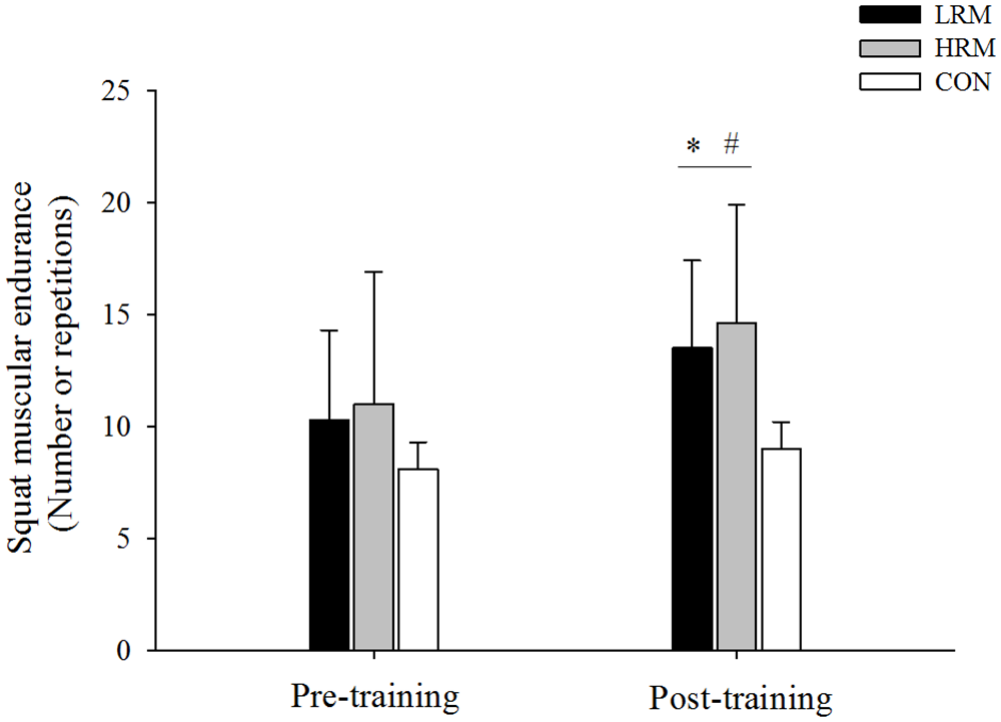
Mean ± SD of squat muscular endurance pre and post 9 week of resistance training. LRM, heavy load–low repetition maximum resistance training group. HRM, moderate load–high repetition maximum resistance training group. CON, control group. (*) p<0.05, higher than pre training. (#) p<0.05, higher than CON group.

There was significant group and time interaction in chest press (F = 3.5, p = 0.039, 1-β = 0.46, Fig 3) and squat muscular strength (F = 3.4, p = 0.042, 1-β = 0.45, Fig 4). Muscular endurance increased significantly for the chest press and squat test in the LRM (*d* = 0.72 and *d* = 1.56, respectively) and HRM (*d* = 0.87 and *d* = 1.46, respectively) groups following resistance training (p< 0.05), with no differences between them. Moreover, squat muscular endurance post-training was higher in the LRM and HRM groups in comparison to the CON group (p<0.05), whereas only chest press muscular endurance post-training for the HRM group was higher than the CON group (p<0.05). The effect size for endurance squat was large in both the LRM (*d* = 1.56) and HRM resistance training (*d* = 1.46) groups. However, for endurance chest press, the effect was large in the HRM resistance training group (*d* = 0.87) and moderate in the LRM resistance training group (*d* = 0.72). Finally, chest press and squat muscular endurance were not altered after 9 weeks in the CON group.

## Discussion

The appropriate prescription of training load and volume is one of the most important challenges when designing a training routine, as they are considered as one of the main variables in youth resistance training [1]. The purpose of this study was to compare the effects of 9 weeks of high-load, low-repetition maximum training with moderate-load, high-repetition maximum resistance training on strength and muscular endurance gains in untrained adolescents. Our first hypothesis was not confirmed; there were no differences in muscular strength between the two resistance training groups. However, our second hypothesis was confirmed, since muscular endurance gains did not differ between both training groups.

Considering that this is the first study to compare the effects of low-repetition maximum and high-repetition maximum resistance training programs on strength and endurance gains in adolescents, the present findings cannot be directly compared to previous studies. However, a variety of resistance training programs can increase muscular strength in youth [1]. Children seem to present similar strength increases in response to high-load and low-load resistance training but exhibit greater endurance after low-load resistance training [23, 24]. It has been verified that high-repetition maximum (15 to 20 RM) resistance training yielded no difference in 1 RM strength gains (21 and 23%, respectively) in children [24]. However, only high-repetition resistance training produced gains in muscular endurance (42%) [24]. Another study found no differences in 1 RM leg extension strength gains in response to 6–8 RM (40.9%) and 13–15 RM (31%) training [23] in children. In contrast, the increase on leg extension muscular endurance was 50.5% greater after 13-15RM training when compared to 6-8RM training [23].

Moreover, several studies in adults found higher maximal muscular force after high-load, low repetition resistance training compared to a low-load, high repetition training regimen [11–17, 34], while muscular endurance gain was optimized after low-load, high-repetition [11–14, 16, 17, 34]. Delorme [11] was the first to show that strength is developed by increasing the resistance load and not by increasing repetitions, whereas muscular endurance improvement is achieved by increasing the number of repetitions. Based on these results, this author emphasized that training regimens aimed at increasing both force and endurance were discrete and that there is no transference between training regimens. Subsequent studies corroborate this assumption, in which greater force after high-load resistance training [11–17, 34] and greater endurance following high-repetition resistance training were reported [11–14, 16, 17, 34]. However, other studies on adults found no differences between high-load, repetition and low-load, repetition and endurance gains [13, 20]. Muscular endurance did not differ among the 6–8 RM vs. 30–40 RM vs. 100–150 RM training regimens [13]. Additionally, Stone and Coulter [20] showed no difference on endurance gains after 6-8RM vs. 15–20 RM and vs. 30-40RM training protocols and these results were similar to the present study. Therefore, the increase (lighter vs. heavier loads) might vary among children, adolescents and adults.

There is the claim that heavier rather than lighter load is required to maximize muscular force development, which was based on the size principle of motor unit recruitment [35]. This principle states that motor units are recruited from smallest to largest and that muscle activation is directly related to the load activity. A greater load resulted in higher motor unit recruitment, which in turn produced greater muscular force. In reality, the size principle strongly suggests that effective resistance training aimed at maximal strength development simply requires the performance of repetitions until failure, regardless of the amount of repetitions (i.e., 4 to 15 RM) [36–38]. However, the results regarding the effectiveness of training until concentric failure for improving neuromuscular adaptation remains controversial. Although some authors have shown some advantages of training until concentric failure in the maximal strength [39] and 6RM strength gains [40], other studies have shown no evidence of a greater magnitude of strength adaptations induced by training until failure [41–43]. In fact, some studies reported an advantage of performing submaximal repetitions per set (i.e., not to failure) in the maximal power output adaptations [42, 44]. Thus, investigating whether these neuromuscular adaptations are related to resistance training to failure (i.e., RM) or to low- and high-load training in the youth population is warranted. However, it is important highlighted that the current stance on youth resistance training recommends heavy loads (>85% 1RM) only after youth achieve a proper exercise technique [1]. Interestingly, although there is a difference in the resting plasma testosterone concentration among pre-adolescent, adolescent, and adults, the strength gains observed in the present study were similar to those in children, while the muscular endurance observed was more similar to that of adults. These findings may be due to the shorter duration (i.e., 9 weeks) of the present study. Thus, studies in adolescents over a longer training duration are important to better understand this topic.

In conclusion, it is generally argued that in resistance training, “heavier is better”. However, the results of the current study suggest that both high-load, low-repetition resistance training and moderate-load, high-repetition resistance training induces similar strength and endurance gains in untrained adolescents. However, the present study is not without limitations. The primary limitation of the current study is that neural adaptation and muscle hypertrophy were not measured, which could help to elucidate the mechanisms related to similar muscle force and endurance in response to LRM and HRM training protocols. Thus, the mechanisms underlying neuromuscular adaptations to strength training in youth require further investigation. From a practical standpoint, the results of the current study suggest that coaches, strength trainers, and athletic trainers can design resistance training ranging from 4 to 15 RM to increase muscular fitness.
